# Plasmacytoid dendritic cell–mediated L-glutamate catabolism links gut microbiota to male infertility

**DOI:** 10.1097/MD.0000000000047440

**Published:** 2026-02-06

**Authors:** Liwen Huang, Xue Li, Yao Hao, Bo Huang, Chengbin Pei, Xiuying Pei, Qian Zhang, Guodong Chen, Shuya Zhang

**Affiliations:** a Key Laboratory of Fertility Preservation and Maintenance of Ministry of Education, School of Basic Medical Sciences, Ningxia Medical University, Yinchuan, China; b Ningxia Basic Medical Research Center, School of Basic Medical Sciences, Ningxia Medical University, Yinchuan, China; c General Hospital of Ningxia Medical University, Yinchuan, China.

**Keywords:** BWMR, gut microbiota, immunometabolism, male infertility, Mendelian randomization, plasmacytoid dendritic cells

## Abstract

Emerging evidence suggests that gut microbiota composition influences male reproductive health; however, the immunometabolic mechanisms underlying this association remain insufficiently characterized. We investigated whether specific immune cell–mediated metabolic pathways, particularly plasmacytoid dendritic cell (pDC)–driven L-glutamate catabolism via the hydroxyglutarate pathway, contribute to the causal link between gut microbiota and male infertility. We conducted a 2-sample, 2-step Mendelian randomization (MR) analysis using inverse-variance weighting as the primary estimator and Bayesian weighted MR for robustness. Exposure data comprised 412 gut microbial taxa/metabolic pathways and 731 immune cell phenotypes from large European-ancestry genome-wide association studies. Male infertility genome-wide association studies data (1429 cases; 128,710 controls) were obtained from FinnGen R10. Only exposure–mediator–outcome pairs meeting stringent pleiotropy, heterogeneity, and reverse-causality criteria were retained for mediation analysis. Nine microbial taxa/metabolic pathways and 18 immune traits exhibited putative causal associations with male infertility. The L-glutamate degradation V pathway via hydroxyglutarate was linked to reduced infertility risk (inverse-variance weighting odds ratio [OR] = 0.68; 95% confidence interval, 0.52–0.89; *P* = .005). Two-step MR suggested that forward scatter area on pDCs may mediate this association, although the mediation effect was imprecise (effect = 0.0277; 95% confidence interval, −0.0348 to 0.0903). This study provides suggestive genetic evidence that pDC-mediated glutamate catabolism may connect gut microbial metabolic activity to male infertility. These findings highlight immunometabolic pathways as testable targets for mechanistic validation and microbiota-directed interventions.

## 1. Introduction

Male infertility represents a substantial contributor to the global infertility burden, with the World Health Organization reporting that approximately 17.5% of adults are affected.^[1]^ Reduced sperm vitality and impaired spermatogenesis are among the most frequent causes, with incidence influenced by both intrinsic and environmental factors.^[2]^

The gut microbiota, a diverse and dynamic microbial ecosystem within the gastrointestinal tract, is integral to nutrient absorption, immune regulation, and metabolic homeostasis.^[3]^ Dysbiosis – an imbalance in microbial composition – has been linked to altered testicular microenvironments, disruption of spermatogenesis, and diminished semen quality.^[4–9]^ Mechanistically, gut microbes metabolize otherwise indigestible proteins and peptides, producing metabolites that regulate immune, metabolic, and neuroendocrine processes.^[10]^

In the male reproductive tract, immune cell populations such as B cells, T cells, monocytes, macrophages, and dendritic cells maintain an immune milieu essential for spermatogenesis and sperm maturation.^[11–16]^ Perturbations to this balance may impair androgen production, weaken immune tolerance to sperm antigens, and trigger inflammatory damage within the testis and epididymis.^[17,18]^ For example, CD8^+^ T cell distribution in the epididymis is critical for pathogen clearance,^[14,19]^ while macrophage depletion in murine models reduces intratesticular testosterone levels.^[17]^ Given the ability of gut microbiota to modulate extraintestinal immunity, a plausible “gut–immune–testis” axis exists. Nevertheless, systematic causal evidence implicating defined microbial metabolic pathways and immune phenotypes as mediators of male infertility remains lacking. We therefore hypothesize the existence of a gut–immune–testis axis in which immune cell–mediated metabolic programs – particularly plasmacytoid dendritic cell (pDC)–driven L-glutamate catabolism – causally link the gut microbiota to male infertility.

Mendelian randomization (MR) uses genetic variants as instrumental variables to infer causal relationships between risk factors and health outcomes. This approach leverages the random allocation of alleles to minimize confounding and reverse causality in observational studies, thereby increasing the reliability of causal inference.^[20,21]^ Compared with randomized controlled trials, MR can circumvent the challenges of large sample sizes and long-term follow-up inherent to interventional studies. Additionally, applying MR to the study of male infertility risk circumvents ethical constraints inherent to randomized trials. Two-step MR extends this approach to evaluate mediators, while Bayesian weighted MR (BWMR) increases robustness under weak instruments and horizontal pleiotropy.^[22]^ Therefore, MR offers a robust study design that addresses key limitations of traditional observational studies and randomized controlled trials.

Here, we employed a 2-sample, 2-step MR and BWMR approach in large genome-wide association studies (GWAS) datasets to examine if immune phenotypes mediate the causal links between gut microbiota features, including metabolic pathways, and male infertility. We focused on pDC-mediated L-glutamate catabolism via the hydroxyglutarate pathway, based on 2 lines of evidence: the distinct immunoregulatory role of pDCs in maintaining testicular immune tolerance, and the critical role of glutamate in cellular energy homeostasis and attenuation of inflammatory responses – processes essential for preserving spermatogenesis in the male reproductive tract.

## 2. Methods

### 2.1. Ethics statement

The GWAS used in this analysis had prior ethical approval from their respective institutional review boards, and all procedures adhered to the Declaration of Helsinki. All datasets are publicly accessible.

### 2.2. Study design

**Figure 1.**
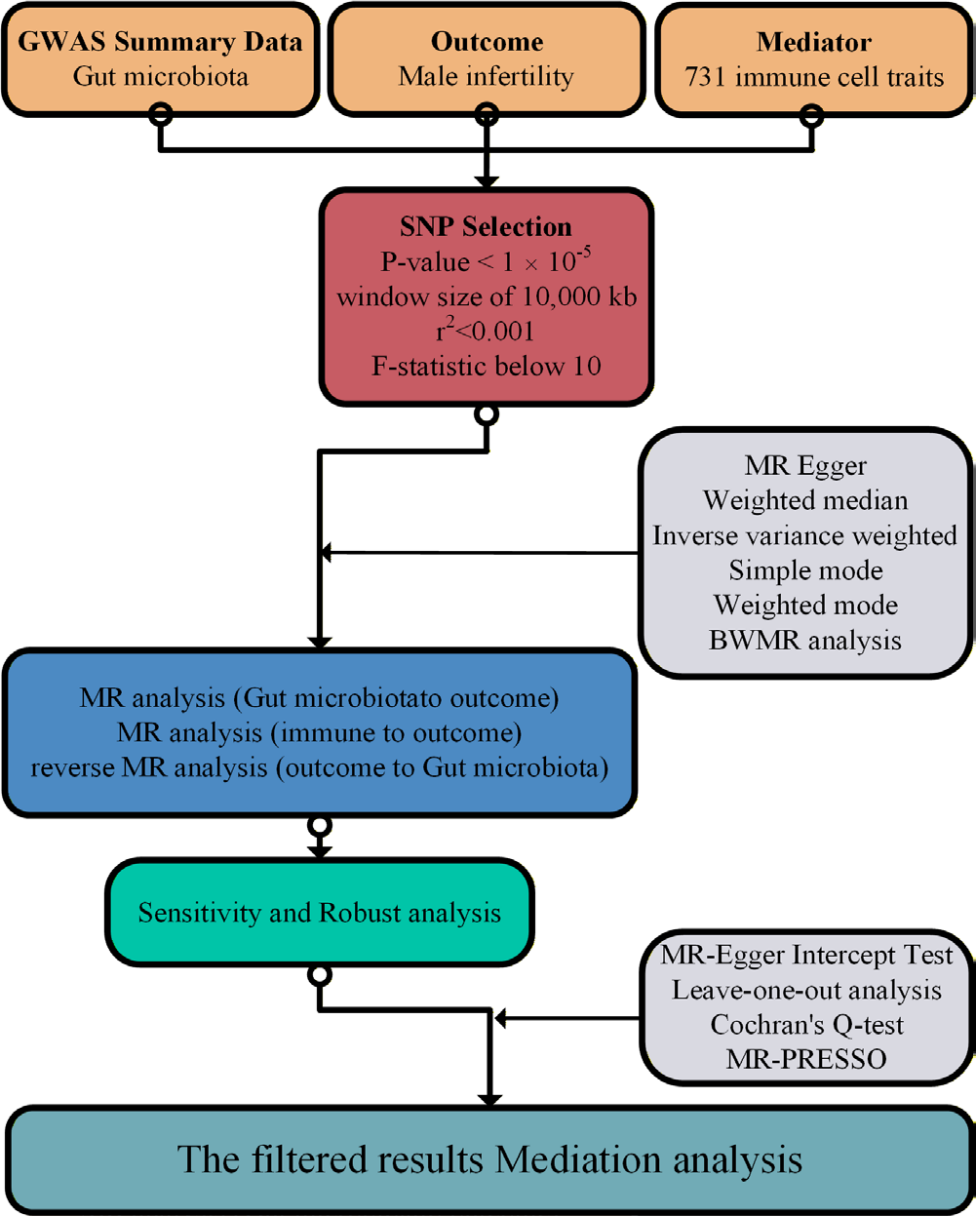
Workflow of the 2-sample, 2-step MR analysis evaluating causal relationships among gut microbiota, immune cell phenotypes, and male infertility. BWMR = Bayesian weighted Mendelian randomization, GWAS = genome-wide association studies, MR = Mendelian randomization, SNP = single-nucleotide polymorphism.

We implemented a 2-sample, 2-step MR framework to examine causal relationships among gut microbiota, immune traits, and male infertility (Fig. 1). Inverse-variance weighting (IVW) was the primary estimator; BWMR was applied for robustness against pleiotropy and weak instruments.

### 2.3. Data sources

#### 2.3.1. Gut microbiota and metabolic pathways

Data on **gut microbiota** were sourced from the Netherlands Microbiome Project, which included a GWAS study involving 7738 participants. The research, led by Lopera-Maya et al, examined 412 gut microbial taxa, including 207 taxa and 205 pathways that reflect microbiome composition and activity. The summary data covered 5 phyla, 10 classes, 13 orders, 26 families, 48 genera, and 105 species.^[23]^

#### 2.3.2. Immune traits

Immune traits data were derived from 731 phenotypes, focusing on median fluorescence intensity (n = 389), absolute cell count (n = 118), relative cell count (n = 192), and morphological parameters (n = 32). These covered various immune cell types, including bone marrow cells, B cells, mature T cells, monocytes, T cells, B cells, natural killer cells, and Treg populations. The data came from a GWAS conducted on 3757 individuals of European ancestry, with no cohort overlap.^[24]^

#### 2.3.3. Male infertility

GWAS data on male infertility were obtained from the FinnGen Consortium (version R10; https://r10.finngen.fi/), which included 1429 cases and 128,710 controls, ensuring a large sample size. All data were sourced from distinct consortia, eliminating any sample overlap.

### 2.4. Instrument selection and harmonization

Single-nucleotide polymorphisms (SNPs) that reached genome-wide significance (*P* < 5 × 10^−8^) were selected as instrumental variables. When fewer than 3 independent instruments were available at this threshold, we relaxed the criterion to *P* < 1 × 10^−5^ to increase instrument availability and strength, a practice commonly adopted in MR studies. Robustness was further evaluated using mendelian randomization using robust adjusted profile score, mendelian randomization pleiotropy residual sum and outlier (MR-PRESSO) with outlier removal, and Steiger filtering to assess causal direction. To ensure independence among instruments, we performed linkage disequilibrium clumping at *r*^2^ < 0.001 within a 10-Mb window.^[25–27]^

Instrument strength was quantified using the *F*-statistic using the formula *F* = *R*² (N − *k* − 1)/[(1 − *R*²)*k*, with values >10 considered adequate. SNP harmonization ensured allele alignment across exposure and outcome datasets.

### 2.5. Statistical analysis

Causal estimates were first generated using IVW Mendelian randomization with the TwoSampleMR, MR-PRESSO, and MendelianRandomization packages in R 4.3.2,^[26,28,29]^ producing odds ratios (ORs) and 95% confidence intervals (CIs) for male infertility across each immune cell type or pathway. To ensure robustness, 6 complementary sensitivity analyses – MR-Egger, weighted median, weighted mode, simple mode, BWMR, and MR-PRESSO ^[22,28,30]^ – were conducted to assess the consistency of direction and magnitude.

Quality control was performed in 3 stages: heterogeneity was assessed using the Cochran *Q* test, where *P* > .05 indicated no significant dispersion^[31]^; horizontal pleiotropy was tested with the MR-Egger intercept and the MR-PRESSO global test. MR-PRESSO also identified and excluded outlying instrumental SNPs contributing to pleiotropy; and leave-one-out analysis was used to evaluate the influence of individual variants on the causal estimates. Any microbial taxon showing residual heterogeneity or pleiotropy after these filters was excluded from further analysis.

Both IVW and BWMR identified positive causal relationships between exposure and male infertility, and this inference was upheld by all robustness checks – MR-Egger intercept, MR-PRESSO distortion test, leave-one-out analysis, and Cochran *Q* test (all *P* > .05).

### 2.6. Mediation analysis

**Figure 2.**
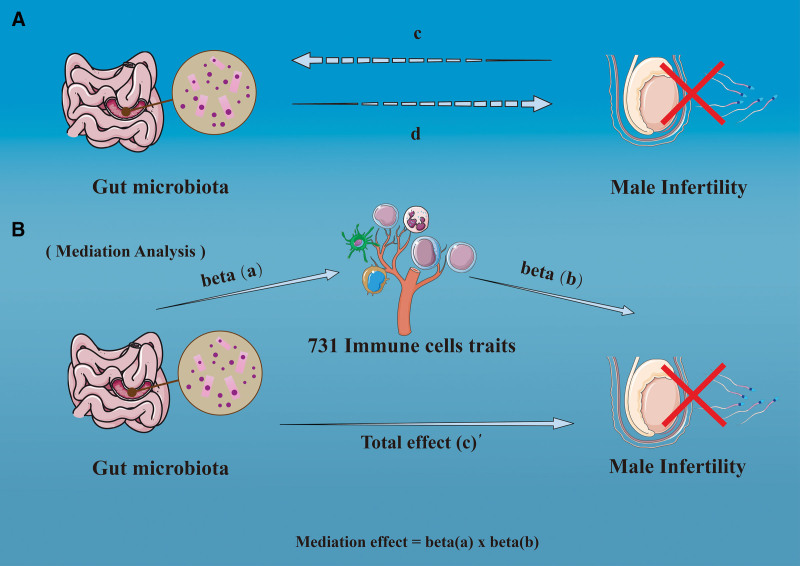
Conceptual diagram of the 2-step MR mediation analysis. (A) The total effect of gut microbiota on male infertility, where *c* represents the total effect using genetically predicted gut microbiota as the exposure and male infertility as the outcome, and *d* represents the total effect in the reverse direction (male infertility as the exposure and gut microbiota as the outcome). (B) The total effect (*c*) was decomposed into the indirect effect and the direct effect. In the 2-step MR approach, *a* denotes the association between gut microbiota and immune cell phenotypes, and *b* denotes the association between immune cell phenotypes and male infertility. The indirect effect was calculated as *a* × *b*, and the direct effect was calculated as *c′* = *c*−(*a* × *b*). MR = Mendelian randomization.

To examine whether immune cell phenotypes potentially mediate the association between gut microbiota features and male infertility, we performed a 2-step mediation analysis within a 2-sample MR framework. In this approach, the total effect of each gut microbiota feature on male infertility was partitioned into 2 components: an indirect effect, representing the portion of the association that operates through the mediator (immune cell phenotype), and a direct effect, representing the portion independent of the mediator^[32]^ (Fig. 2).

The indirect effect was calculated by multiplying the effect estimate from the exposure to the mediator by the effect estimate from the mediator to the outcome. The direct effect was obtained by subtracting the indirect effect from the total effect. To quantify uncertainty, we used the delta method to calculate 95% CIs for the mediation estimates and conducted additional bootstrap analyses to assess robustness.^[33]^

## 3. Results

### 3.1. Gut microbiota metabolic pathways linked with male infertility

IVW-MR identified 9 gut microbial taxa or metabolic pathways with putative causal associations with male infertility after false discovery rate correction. Four pathways were associated with reduced risk, with the L-glutamate degradation V pathway via the hydroxyglutarate route showing the strongest protective association (IVW OR = 0.68; 95% CI, 0.52–0.89; *P* = .005). Conversely, 5 pathways were associated with increased risk, the most pronounced being palmitate biosynthesis II (bacteria and plants) (IVW OR = 1.51; 95% CI, 1.16–1.97; *P* = .002) (Table 1A).

**Table 1:**
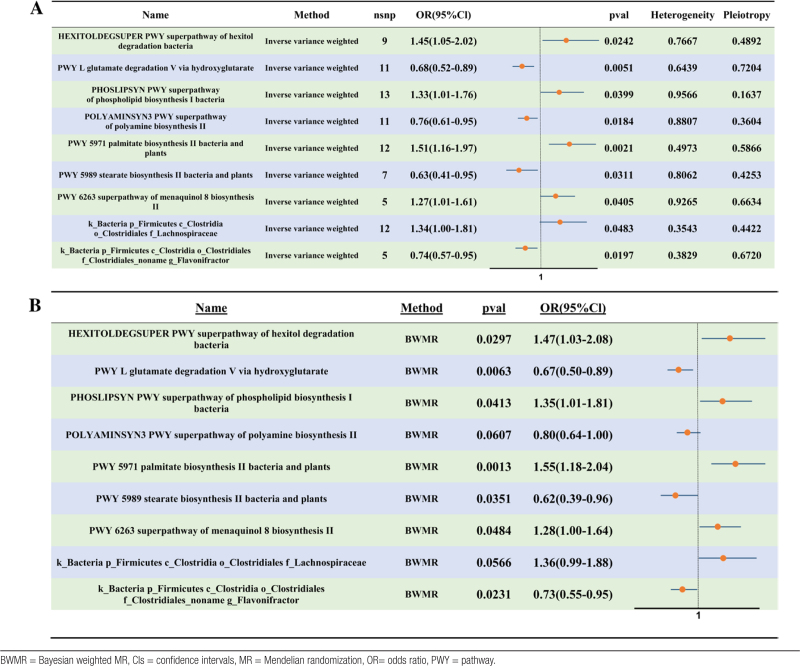
Gut microbiota metabolic pathways significantly associated with male infertility in the IVW-MR analysis (A) and BWMR analysis (B).

BWMR confirmed 7 of the 9 IVW-identified pathways, with polyamine biosynthesis II (POLYAMINSYN3) and Lachnospiraceae abundance excluded due to borderline (*P* > .05) (Table 1B).

### 3.2. Gut immune cells linked with male infertility

IVW-MR identified 30 immune cell phenotypes with putative causal associations with male infertility, comprising 11 phenotypes associated with increased risk and 19 with reduced risk. Among the risk-associated traits, CD38 expression on IgD^+^ B cells had the strongest effect (IVW OR = 1.20; 95% CI, 1.04–1.39; *P* = .0027). Among protective traits, the morphological parameter forward scatter area (FSC-A) on pDCs was the most significant (IVW OR = 0.91; 95% CI, 0.85–0.97; *P* = .0002; Table 2A).

**Table 2:**
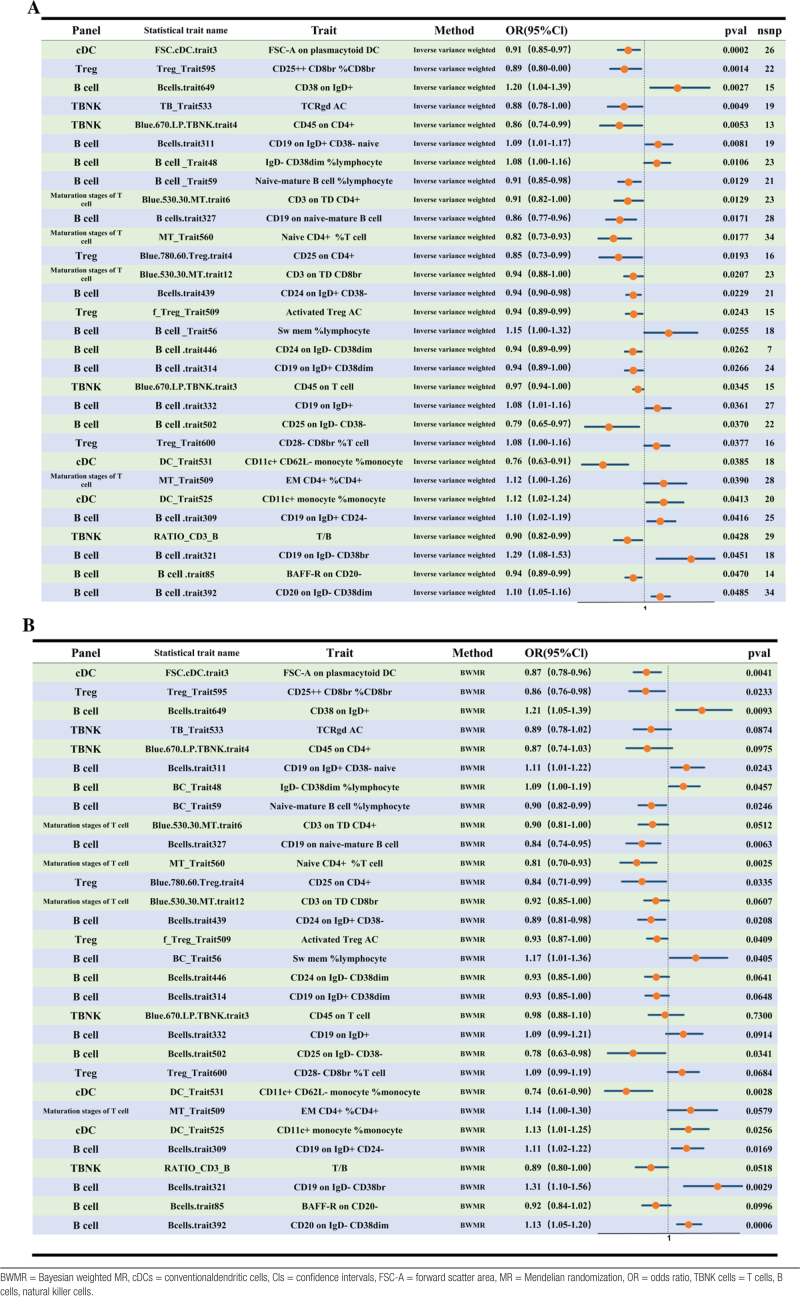
Immune cell phenotypes significantly associated with male infertility in the IVW-MR analysis (A) and BWMR analysis (B).

BWMR confirmed associations for 18 of the 30 IVW-identified phenotypes, excluding 12 traits such as total T cells, B cells, natural killer cell counts, and γδ T cell absolute counts (*P* > .05) (Table 2B).

Reverse IVW-MR using male infertility as the exposure found no significant effects on gut microbial taxa or pathways previously identified in forward analysis (Table 3A). Reverse BWMR also detected no significant effects, except for menaquinone-8 biosynthesis II, which showed a small but statistically significant association (OR = 0.87; 95% CI, 0.77–0.98; *P* = .0275), warranting cautious interpretation (Table 3B).

**Table 3:**
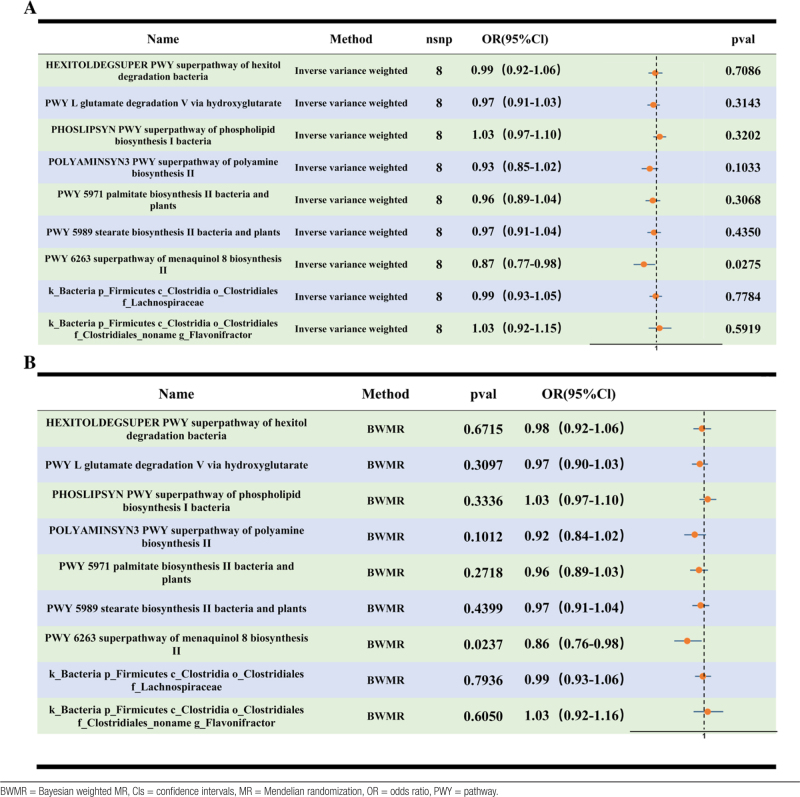
Associations of male infertility with gut microbiota metabolic pathways in the IVW-MR analysis (A) and BWMR analysis (B).

### 3.3. Mediation analysis

Two-step MR suggested that FSC-A on pDCs may mediate the association between the L-glutamate degradation V pathway via hydroxyglutarate and male infertility. The indirect effect was small and imprecise (effect = 0.0277; 95% CI, −0.0348 to 0.0903), indicating considerable uncertainty. (Table 4).

**Table 4:** Mediation effect of FSC-A on plasmacytoid dendritic cells in the association between L-glutamate degradation V via the hydroxyglutarate pathway and male infertility.

Mediator	The effect of exposure on outcome β (95% CI)	The effect of exposure on mediator β1 (95% CI)	The effect of mediator on outcome β (95% CI)	Mediator effect (95% CI)
FSC-A on plasmacytoid cDC	−0.3779 (−0.6425, −0.1133)	0.2893 (0.0736, 0.5050)	0.0957 (0.0453, 0.1461)	0.0277 (−0.0348, 0.0903)

cDCs = conventional dendritic cells, CIs = confidence intervals , FSC-A = forward scatter area.

### 3.4. Sensitivity analyses

No substantial horizontal pleiotropy or significant heterogeneity was detected in MR-Egger intercept tests, Cochran *Q* statistics, or MR-PRESSO global tests. Leave-one-out analyses indicated that individual SNPs did not disproportionately influence the causal estimates (Table 5).

**Table 5:** Sensitivity analysis results for the MR estimates, including the MR-Egger intercept test, Cochran *Q* test, and MR-PRESSO global test.

Exposure	Outcome	Heterogeneity_MR Egger	Heterogeneity_IVW	Pleiotropy
L-glutamate degradation V via hydroxyglutarate	Male infertility	0.563651491	0.643868654	0.72038772
L-glutamate degradation V via hydroxyglutarate	FSC-A on plasmacytoid DC	0.928388765	0.952686188	0.707564387
FSC-A on plasmacytoid cDC	Male infertility	0.347904416	0.32150078	0.236524582

cDCs = conventional dendritic cells, FSC-A = forward scatter area, IVW = inverse-variance weighted, MR = Mendelian randomization, MR-PRESSO = mendelian randomization pleiotropy residual sum and outlier.

## 4. Discussion

The gut microbiota plays a central role in regulating host immune function. Dysbiosis of the gut microbiota may impair male reproductive function by triggering immune-mediated inflammation that damages the epididymis and downregulates genes critical for spermatogenesis.^[8,34,35]^ For example, Ding et al demonstrated that in high-fat diet–fed mice, microbial imbalance leads to excessive endotoxin-driven immune activation, elevating pro-inflammatory factors in the epididymis and reducing sperm viability.^[9]^ Guiton et al reported that dietary fiber supplementation can beneficially modulate gut microbiota composition, thereby supporting spermatogenesis and improving semen quality.^[36]^ However, the immunometabolic mechanisms linking gut dysbiosis to male infertility remain poorly defined.

This study integrates large-scale GWAS data to evaluate the potential causal role of gut microbiota in male infertility and the mediating influence of immune cell phenotypes. Using IVW and BWMR methods, we identified 18 immune cell traits and 9 gut microbiota taxa or pathways with putative causal associations with male infertility. Our findings suggest that the L-glutamate degradation V pathway via hydroxyglutarate may influence male infertility through FSC-A–related phenotypes in pDCs. However, the observed mediation effect of pDC FSC-A was imprecise, as the 95% CI included zero. Accordingly, this finding should be considered hypothesis-generating and warrants independent validation. Collectively, these findings provide insights into immunometabolic pathways in male infertility and suggest potential targets for microbiota- or metabolism-directed interventions.

Our findings support the hypothesis of a “gut–immune–testis” axis, whereby microbial metabolism may influence male reproductive outcomes through immune-mediated mechanisms. Glutamate metabolism is integral to protein synthesis, cellular energy balance, and immune regulation.^[37–40]^ In the reproductive context, glutamate and aspartate have been shown to mitigate inflammatory damage in the testes and epididymis, partly via upregulation of transforming growth factor-β and interleukin-10.^[40,41]^

pDCs are potent antigen-presenting cells with unique immunoregulatory functions.^[42–45]^ In the male reproductive tract, immature pDCs contribute to immune tolerance toward sperm antigens, whereas their maturation under pathological conditions can trigger autoimmune responses detrimental to spermatogenesis.^[46]^ Our mediation analysis suggests that morphological variation in pDCs (as captured by FSC-A) could partially account for the microbiota–infertility association, although effect estimates lacked precision. Interpretation of this finding warrants caution, because FSC-A is a flow-cytometric parameter that primarily reflects cell size, and to a lesser extent internal complexity, rather than a direct measure of pDC function (e.g., antigen-presentation capacity or cytokine secretion). Although our genetic epidemiologic analysis supports a putative causal link and implicates pDC-related biology, the mechanism by which L-glutamate degradation influences pDCs or spermatogenesis remains unresolved. Metabolites within the glutamate pathway, such as α-ketoglutarate,^[47]^ can modulate immune cell differentiation and function through epigenetic regulation and redox homeostasis. Building on prior literature, it is plausible that these metabolites reshape the testicular immune microenvironment, for example, by altering inflammatory cytokine balance or signaling pathways such as transforming growth factor-β that help maintain immune privilege in the reproductive tract.^[48]^ Nevertheless, the functional impact of glutamate catabolism on pDC activity and the downstream consequences for spermatogenesis remain hypotheses that require direct experimental testing in vitro and in vivo.^[49]^ Accordingly, while our genetic epidemiologic analysis points to a pDC-related component in the link between the gut microbiota and infertility, the operative mechanisms must be elucidated through targeted experimental studies. From a translational standpoint, identifying immunometabolic mediators provides a framework for targeted interventions. For example, dietary modulation of glutamate metabolism or microbiota-directed therapies (e.g., probiotics, prebiotics) could be explored as adjunctive strategies in managing male infertility. However, such applications require experimental validation and careful consideration of systemic metabolic effects.

Several limitations merit consideration. First, all GWAS datasets were derived from populations of European ancestry, which limits generalizability to other ancestries. Replication in diverse cohorts, for example, East Asian and African populations, is essential. Second, although MR reduces confounding and reverse causality, it cannot exclude residual horizontal pleiotropy or bias from unmeasured confounders that may influence both the genetic instruments and the outcome. Factors such as long-term diet, specific environmental exposures, or socioeconomic status could act as such confounders. Although sensitivity analyses were used to probe pleiotropy, the influence of these factors cannot be entirely ruled out. Finally, mediation effect estimates involving pDC traits were imprecise, and the causal direction of these associations requires further investigation. Moreover, this study relied solely on genetic data; no functional assays or experimental validation in vitro or in vivo were conducted to verify the biological mechanisms underlying the observed associations. Accordingly, our findings should be interpreted as highlighting putative causal pathways that require mechanistic confirmation in future studies.

## 5. Conclusion

Using 2-sample, 2-step MR and BWMR approaches, we provide suggestive evidence linking specific gut microbial pathways – particularly L-glutamate degradation via hydroxyglutarate – to reduced male infertility risk, potentially mediated by pDC morphological traits. These findings highlight immunometabolic pathways as promising targets for mechanistic research and potential microbiota-based interventions. Future studies should integrate genetic epidemiologic evidence with mechanistic studies in immunology and reproductive biology to validate these associations. Ultimately, integrated approaches, including in vitro and in vivo models, are needed to elucidate the underlying biological mechanisms and to evaluate the therapeutic potential of targeting these pathways for the treatment of male infertility.

## Acknowledgments

GWAS summary data were downloaded from the public platform (https://gwas.mrcieu.ac.uk/). We want to acknowledge the participants and investigators of the FinnGen study.

## Author contributions

**Conceptualization:** Liwen Huang, Bo Huang, Chengbin Pei, Xiuying Pei, Qian Zhang, Shuya Zhang.

**Data curation:** Liwen Huang, Xue Li, Bo Huang, Qian Zhang.

**Visualization:** Liwen Huang, Xue Li.

**Software:** Shuya Zhang.

**Formal analysis:** Xue Li.

**Supervision:** Xiuying Pei.

**Writing – original draft:** Liwen Huang, Yao Hao.

**Writing – review & editing:** Xiuying Pei, Guodong Chen, Shuya Zhang.
